# Virulence of Bacteria Causing Mastitis in Dairy Cows: A Literature Review

**DOI:** 10.3390/microorganisms13010167

**Published:** 2025-01-15

**Authors:** Xiaofang Tong, Herman W. Barkema, Diego B. Nobrega, Chuang Xu, Bo Han, Chenyibo Zhang, Jingyue Yang, Xiaoping Li, Jian Gao

**Affiliations:** 1 College of Veterinary Medicine, China Agricultural University, Beijing 100193, China; xxf6767@163.com (X.T.); xuchuang@cau.edu.cn (C.X.); hanbo@cau.edu.cn (B.H.); zcyb@cau.edu.cn (C.Z.); yangjingyue0303@126.com (J.Y.); xiaopingli0512@163.com (X.L.); 2 Faculty of Veterinary Medicine, University of Calgary, Calgary, AB T2N 4N1, Canada; barkema@ucalgary.ca (H.W.B.); diego.nobrega@ucalgary.ca (D.B.N.)

**Keywords:** intramammary infection, virulence properties, mastitis pathogens, bovine mastitis, virulence factor

## Abstract

Bovine mastitis, a prevalent disease in dairy farms, exerts a profound negative influence on both the health and productivity of dairy cattle, leading to substantial economic losses for the dairy industry. The disease is associated with different bacterial agents, primarily Gram-positive cocci (e.g., *Staphylococcus* spp., *Streptococcus* spp.) and Gram-negative bacilli (e.g., *Escherichia coli*, *Klebsiella pneumoniae*). These pathogens induce mastitis through diverse mechanisms, intricately linked to the virulence factors they carry. Despite previous research on the virulence factors of mastitis-causing bacteria in dairy cattle, there remains a significant gap in our comprehensive understanding of these factors. To bridge these gaps, this manuscript reviews and compiles research on the virulence factors of these pathogens, focusing on their roles in mammary tissue infection, immune evasion, adherence to mammary epithelial cells, and invasion and colonization of the mammary gland. These processes are analyzed in depth to provide a comprehensive framework to promote a deeper understanding of dairy pathogenic bacteria and their pathogenic mechanisms and to provide new insights into the control of mastitis in dairy cattle.

## 1. Introduction

**Figure 1:**
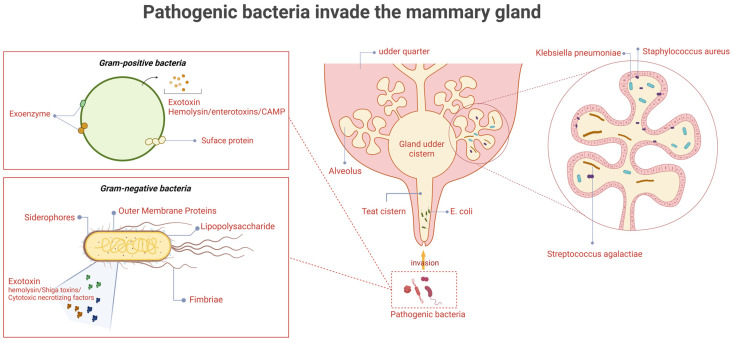
The invasion of pathogenic bacteria in the mammary gland. The causative organisms first enter through the teat canal, followed by the teat cistern, gland cistern, and ducts, before finally reaching the alveoli. Gram-positive organisms (e.g., *Staphylococcus aureus* and *Streptococcus agalactis*) exert their pathogenic effects primarily through surface proteins and exotoxins such as haemolysins or CAMP factors. In contrast, Gram-negative organisms (e.g., *Escherichia coli* and *Klebsiella pneumoniae*) mainly act via fimbriae, outer membrane proteins, lipopolysaccharides, and iron transport systems. (Created with BioRender.com).

Bovine mastitis is one of the costliest diseases on dairy farms, resulting in decreased milk production, decreased reproductive capacity, loss of discarded milk, and increased treatment costs [1]. Mastitis in dairy cows is caused by a wide variety of pathogens, including Gram-positive cocci, Gram-negative bacilli, *Mycoplasma bovis*, and the *Prototheca species* [2]. Among these, Gram-positive cocci and Gram-negative bacilli are the main groups of pathogenic bacteria. These pathogens are capable of producing various virulence factors that facilitate their colonization and infection in the mammary tissue of dairy cows. Mastitis-derived Gram-positive cocci include *Staphylococcus* spp. and *Streptococcus* spp., which produce virulence factors such as exotoxins, enzymes, and surface proteins, whereas Gram-negative bacilli such as *Escherichia coli* and *Klebsiella pnenmoniae* produce lipopolysaccharides, iron carriers, exotoxins, and outer membrane proteins to increase their pathogenicity (Figure 1). In addition, *Staphylococcus aureus* and *Streptococcus agalactiae* can survive in the mammary gland tissue of mastitis cows for a long period, being shed in milk and spreading to healthy cows through milking machine liners, milker’s hands and towels, resulting in cow-to-cow transmission [3]. *E. coli*, *K. pneumoniae*, and environmental streptococci (*Strep. uberis*, *Strep. dysgalactiae*) mainly live in cow’s manure, bedding, barn floor, slurry, etc., and usually enter the udder through the teat duct when the cow is lying down [4]. Coagulase-Negative Staphylococci (CNS) are mainly found in the teat skin and teat duct of cows and are mostly opportunistic pathogens, usually causing subclinical mastitis or mild clinical mastitis [5].

**Figure 2:**
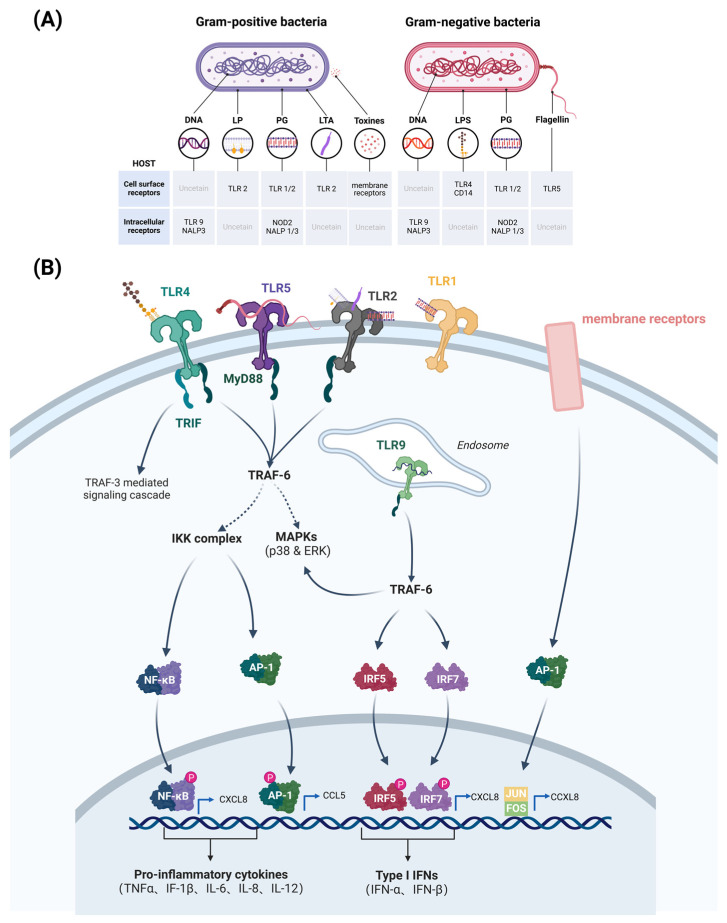
Pathogen-associated molecular patterns of pathogenic bacteria that correspond to receptors (**A**), and pathogenic bacteria that activate major signaling pathways of bovine mammary epithelial cells (bMECs) to produce pro-inflammatory cytokines and chemokines (**B**). Gram-negative PAMPs bind mainly to TLR4 receptors, triggering the NF-κB pathway and producing pro-inflammatory cytokines and chemokines, such as IL-1β, IL-6, IL-8, and TNF-α. Also, the expression of type I interferon genes, such as IFN-β, is induced. On the other hand, TLR2 and membrane receptors recognize the peptidoglycan of the Gram-positive bacterial cell wall, positive bacterial cell wall peptidoglycan components and toxins, activating NF-κB and AP-1 signaling pathways. TLR9 in mammary epithelial cell endosomes recognizes unmethylated CpGDNA in bacterial DNA, activating downstream signalling pathways, including the MyD88-dependent pathway and TRAF-6-dependent pathway. (Created with BioRender.com).

Bovine mammary epithelial cells (bMECs) are a major target for infection by pathogenic bacteria. Virulence factors of pathogenic bacteria often stimulate Toll-like receptors (TLRs) or nucleotide-binding oligomerization domain (NOD)-like receptors on bMECs and activate the innate immune system in the mammary gland of cows. These receptors mediate the immune response by recognizing pathogen-associated molecular patterns (PAMPs) present in the bacterial cell wall or cytoplasm, such as lipopolysaccharides (LPSs), lipoprotein (LPs), peptidoglycan (PG), lipoteichoic acid (LTA), toxins, and flagellin (Figure 2A) [6]. Gram-negative bacilli (e.g., *E. coli*) can activate the Toll-like receptor 4 (TLR4) signaling pathway in bMECs. TLR4 interacts with LPS components of the cell walls of Gram-negative bacteria, thereby triggering the activation of NF-κB and mitogen-activated protein kinase (MAPK) signaling pathways (Figure 2A) [7]. This activation results in the upregulation of pro-inflammatory cytokines, including IL-1β, IL-6, IL-8 and TNF-α, which in turn facilitates the recruitment of immune cells to the site of infection for sterilization (Figure 2B) [7]. In contrast, Gram-positive bacteria (e.g., *Staph. aureus*) activate the Toll-like receptor 2 (TLR2) signaling pathway in bMECs. TLR2 and membrane receptors recognize peptidoglycan components and toxins in the bacterial cell wall, respectively, activating the NF-κB and protein 1 (AP-1) signaling pathways (Figure 2A), leading to the release of IL-1β, IL-6, CXCL8 and CCL5 (Figure 2B) [7]. In addition, pathogenic bacteria can act on TLR9 of bMECs to trigger an immune response. TLR9 is located in the endosome and recognizes the unmethylated CpG pattern in bacterial DNA (Figure 2A). Stimulation of TLR9 by pathogenic bacteria leads to activation of downstream signaling pathways (MyD88-dependent pathway and TRAF-6-dependent pathway), resulting in the production of Type I interferons (IFNs) and pro-inflammatory cytokines that mediate the bactericidal effect of immune cells (Figure 2B).

Virulence factors play a pivotal role in augmenting the mammary pathogenicity of bacteria and exhibit a strong correlation with the inflammatory response within the mammary glands of dairy cattle [8]. Over the years, researchers have studied the distribution of virulence factors or pathogenicity mechanisms of mastitis pathogenic bacteria. These studies have provided important information about the key virulence characteristics of those major pathogenic bacteria and to uncover their pathogenic mechanisms. This review summarizes the current understanding of key virulence factors of mastitis pathogens and their contribution to the pathogenesis of mastitis. The information described herein offers novel perspectives for further research on the pathogenesis of mastitis and the development of control strategies, diagnostic tools, vaccines and therapeutic agents.

## 2. *Staphylococcus aureus*

**Figure 3:**
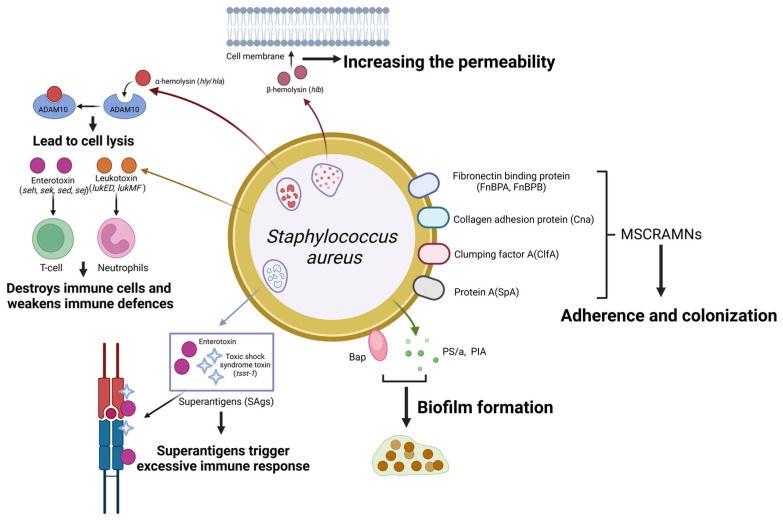
Schematic representation of *Staphylococcus aureus* virulence factors and their roles in mastitis pathogenesis. *Staph. aureus* expresses a range of cell surface proteins termed MSCRAMMs that enhance its adherence to and colonization of host tissues. These include fibronectin-binding proteins (encoded by *FnBPA* and *FnBPB*), collagen adhesion protein (encoded by *Cna*), clumping factors (encoded by *clfA*), and protein A (encoded by *SpA*). α-hemolysin interacts with ADAM10, and β-hemolysin enhances cell membrane permeability, contributing to cell lysis. Superantigens (SAgs), including enterotoxins and toxic shock syndrome toxin-1 (TSST-1), elicit exaggerated immune responses. In contrast, leukotoxins (encoded by *LukED* and *LukMF*) target and destroy immune cells, thereby weakening the host’s immune defences. *Staphylococcus aureus* also secretes Bap, PS/A, and PIA, which promote biofilm formation, making survival easier in environments. (Created with BioRender.com).

*Staph. aureus* is a species of Gram-positive, peroxidase- and coagulase-positive, cluster-forming, non-motile and non-spore-forming facultative anaerobic bacterium [9], mainly spreading between cows during milking. It can cause clinical mastitis, yet it more commonly leads to subclinical and often chronic forms of mastitis, accompanied by an elevated milk SCC [10]. Many kinds of virulence factors that enhance bacterial survival and proliferation within the mammary gland are proven to be excreted by mastitis-derived *Staph. aureus* (Figure 3). These virulence factors are mostly regulated by a quorum-sensing accessory gene regulator (agr) system and their abundance correlates with bacterial virulence and pathogenicity [11].

### 2.1. Adherence

Adhesion to bMECs is thought to be a key pathogenic mechanism in *Staph. aureus*-derived mastitis [12]. *Staph. aureus* utilizes a range of microbial surface components recognizing adhesive matrix molecules (MSCRAMNs) to colonize bMECs, including fibronectin-binding protein A (FnBPA), fibronectin-binding protein B (FnBPB), clumping factor A (ClfA), collagen adhesion (Cna) proteins and Protein A (SpA) [13,14]. MSCRAMMs are cell-wall-anchoring proteins with common structural features, including an N-terminal folding structural domain involved in ligand binding and a sorting signal located at the C-terminus that anchors the protein to the cell wall [15].

In bovine mammary gland, *Staph. aureus* adhesion and invasion are promoted mainly by FnBPA and FnBPB. Experimental mutant strains lacking FnBPs were shown to have lower colonization capacity in mouse mammary glands, confirming that FnBPs have a competitive advantage in vivo and can be considered as virulence factors for mammary gland colonization [16]. Overexpression of the *fnbB* gene, encoding for FnBPB, has been associated with an enhanced invasive capacity towards bMECs [12]. However, the invasive capacity of the bacteria was retained despite the absence of these genes, suggesting that other factors may be involved in this process [17]. It is noteworthy that the *clfA* gene is highly prevalent (63.7–100%) in *Staph. aureus* isolates from dairy cows across different countries [18]. Research indicates that clfA adheres to bMECS in a fibrinogen-independent manner, mediated by the membrane-bound protein A2 receptor [13].

### 2.2. Exotoxins

When *Staph. aureus* adheres to bMECs, it releases exotoxins that destroy tissue and cause inflammation, including hemolysins, enterotoxins, toxic shock syndrome toxin 1 (TSST-1), and leukotoxins [19]. *Staph. aureus* commonly produces α- and β-hemolysins. α-hemolysin (Hla) binds to the cell membrane receptor ADAM10, leading to an increase in intracellular calcium (Ca^2+^) levels and a rapid decrease in potassium (K^+^) levels. A study has found that 14 (64%) of 22 single nucleotide mutations in the *hla* gene encoding the α-hemolysin in mastitis-derived *Staph. aureus* were identical to human isolates [20], suggesting that transmission of *Staph. aureus* may occur between dairy cows and humans, which would be of great relevance to public health security. β-hemolysin, although not causing cell lysis, acts as a sphingomyelinase, increasing the permeability of the host cell and cell surface charge progressive loss, making the cells more susceptible to the action of α-hemolysin [21].

*Staph. aureus* enterotoxins are orchestrated by multiple genes, and most clinical isolates of bovine mastitis contain one or more enterotoxin genes. This variation in distribution and frequency was associated with the clinical manifestations of the infection. It was shown that seh and sek were more prevalent in isolates causing subclinical mastitis, while sed and sej were mainly associated with persistent mastitis [22]. Enterotoxins M and H contribute to inflammation, necrosis and apoptosis of bMECs [23,24], and pathology was also observed when staphylococcal enterotoxin C (SEC) was injected into the mammary gland of mice [25]. However, the high concentrations of toxin used in these studies may not fully mimic what happens during in vivo infection and may play a limited role during nature intramammary infection.

Enterotoxin and TSST-1 are superantigens (SAgs) of *Staph. aureus* that stimulate the release of bovine T cells and pro-inflammatory cytokines, including interleukin-1 (IL-1), IL-2, IL-6, tumor necrosis factor α (TNF-α), gamma interferon (IFN-γ) and chemokines CCL 2 and CCL 3 [26,27]. It has been shown that most superantigens (SAg) are produced by *Staph. aureus* strain RF122, which is mitogenically active against bovine T cells at low concentrations [27]. This may suggest that at low concentrations in the early stages of infection, SAg may have an impact on the development and persistence of bovine, highlighting the functional importance of SAgs during bovine infection.

Different leukocidin variants have been demonstrated in strains of bovine origin, such as lukS/lukF (γ-hemolysin), lukD/lukE, and especially the bi-component leukocidin lukMF, which plays an important role in the establishment of infections [28,29]. The lukMF’ toxin was only detected in *Staph. aureus* strains associated with animals such as cattle and sheep [30]. Later, it was shown that the expression of CCR1 in cattle, but not on human neutrophils, was responsible for the species-specific killing of lukMF’ [9]. For mastitis control programs, detection of the *lukMF* gene in dairy farms helps to identify strains of *Staph. aureus* adapted to cattle and became an additional tool.

It is worth noting that toxins may still partly remain biologically and immunogenically active even after pasteurization and/or food processing [19]. Therefore, stringent control measures must be implemented to prevent *Staph. aureus* from contaminating milk with exotoxins. This is crucial for safeguarding the quality of milk and dairy products, ultimately serving to protect human health.

### 2.3. Biofilms

The ability of *Staph. aureus* to form biofilms is well known, allowing the bacteria to establish and maintain a protected niche, thereby developing resistance to antimicrobials and environmental stressors [31]. The formation of *Staph. aureus* biofilm involves a variety of proteins and genes, including capsular polysaccharide/adhesin (PS/a) and polysaccharide intercellular adhesins (PIAs) [32]. Studies have shown that the ica locus (icaABCD), which synthesizes PS/s and PIA, is highly prevalent in *Staph. aureus* isolated from bovine mastitis [32]. However, other research has found that interruption of the *ica* operon of *bap*-positive *Staph. aureus* strains isolated from bovine mastitis did not affect biofilm formation in vitro, suggesting that the surface protein Bap compensates for the absence of PIA/PNAG(poly-β (1–6)-N-acetylglucosamine) products [33]. There may also be other undiscovered biofilm formation-related factors that may be involved in or contribute to this compensatory mechanism, and this potential mechanism deserves further in-depth investigation.

At lower concentrations of calcium, Bap is cleaved into fragments that form amyloid fibrils that provide a scaffold for biofilm development [34]. These data suggest that bovine-derived *Staph. aureus* forms biofilms in a PIA-dependent manner during lactation, but the PIA-independent mechanism via Bap may play a role during the dry cow period when calcium concentrations in the udder are low. Bap was also found to facilitate the formation of bacterial aggregates attached to bMECs by weakening bacterial internalization through interaction with Gp96 expressed in mammary epithelial cells [35]. In this context, the Bap biofilm matrix promotes the establishment of long-term persistent infection and mediates immune evasion by masking surface antigens [35].

Biofilm production and PNAG in *Staph. aureus* have been linked to increased mastitis persistence within herds, as well as reduced cytotoxicity and high invasiveness [36]. Despite the clear role of biofilms in immune evasion and chronic infection, the role of biofilms in mastitis pathogenesis remains controversial. One study found that the ability of *Staph. aureus* to invade bMECs did not differ significantly between biofilm-producing and non-biofilm-producing strains [37]. Meanwhile, Pereyra et al. (2016) found that biofilm production was not associated with *Staph. aureus* internalization of bMECs [12]. Both source clinical non-persistent intramammary infections (NP IMIs) and subclinical persistent intramammary infections (PIMIs) were internalized in bMECs (MAC-T) and there was no difference in the ability of these strains to internalize. None of the *Staph. aureus* strains in the study possessed the *bap* gene [12]. Other in vitro studies have shown that *Staph. aureus* biofilms had a lower ability to invade bMECs compared to planktonic *Staph. aureus* cultures and biofilm cultures induced less cell activation than planktonic cultures [38]. These findings are in contrast to studies by Buzzola et al. (2007) and Bardiau et al. (2014) who reported an association between biofilm formation and increased invasiveness of MAC-T [39,40]. These inconsistencies may stem from differences in the *Staph. aureus* strains involved in the studies. It may also stem from differences in experimental conditions and study methods, with Pereyra et al. (2016) [12] (multiplicity of infection, MOI ≈ 100) using a higher MOI than Bardiau et al. (2014) [40] (MOI ≈ 40) and Buzzola et al. (2007) [39] (MOI = 10 to 40). Therefore, further studies are needed to elucidate the exact role of biofilms in the pathogenesis of mastitis.

## 3. *Streptococcus agalactiae*

**Figure 4:**
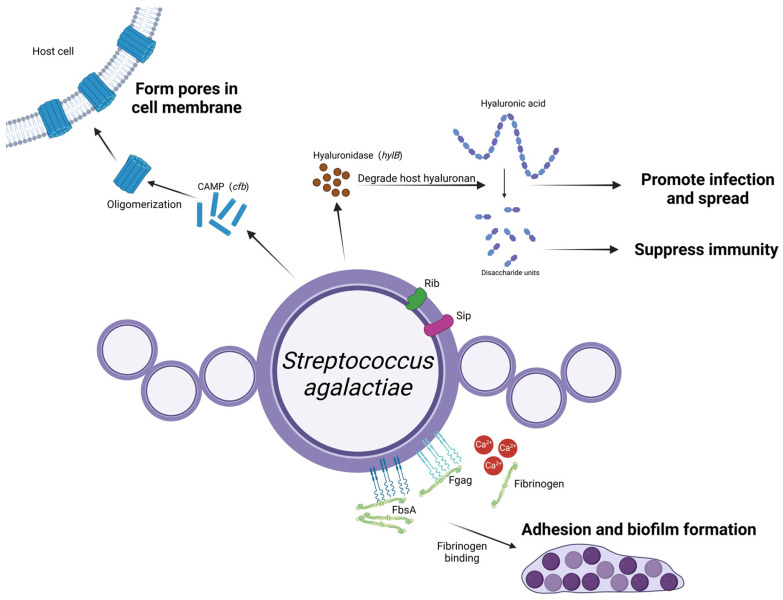
Schematic representation of *Streptococcus agalactiae* virulence factors and their roles in mastitis pathogenesis. *Strep. agalactiae* expresses FbsA and Fgag on its surface, promoting biofilm formation and adherence to epithelial cells within the mammary gland, and the high calcium concentration in the mammary gland enhances the interaction between Fgag and fibrinogen. Upon entering the mammary gland, *Strep. agalactiae* secretes *hylB*-encoded hyaluronidase that degrades hyaluronan. The resulting disaccharide units suppress the host’s immune response, facilitating deeper bacterial invasion. CAMP factors, encoded by the *cfb* gene, oligomerize after being excreted from the bacteria, forming tube-like structures that create pores in the host cell membrane and in turn cause cell damage. Other surface proteins, including Rib and Sip, require further investigation to determine their roles. (Created with BioRender.com).

*Strep. agalactiae* (group B *streptococcus*; GBS) is the causative agent of contagious mastitis and most infected cattle do not show clinical signs, but its effect on milk production is severe and accompanied by a very high somatic cell count (SCC) [41]. When *Strep. agalactiae* infects a cow’s udder, it stimulates innate immunity of the cow’s mammary gland and regulation of virulence factors at every stage after infection [42], including proteins that allow it to adhere, colonize, and invade host cells (fibrinogen-binding protein and Rib protein), toxins (β-hemolysin and CAMP factors), and factors that can evade host recognition (c5a enzyme and hyaluronidase). These virulence factor types, their encoding genes and their role in infecting the mammary gland of cows are illustrated in Figure 4.

### 3.1. Adherence

Fibrinogen (Fg) is a host extracellular matrix component (ECM). In *Strep. agalactiae*, the two surface proteins that recognize Fg are FbsA and FbsB [43]. FbsB, which originates from human *Strep. agalactiae*, has a homologous protein in bovine *Strep. agalactiae* called Fgag [44]. The N-terminal region of FbsB binds to human Fg, whereas the C-terminal region of Fgag (FbsB(C)) binds to bovine Fg [44]. Ca2^+^ ions enhance the adhesion of FbsB(C) proteins, a factor that may explain the tendency of *Strep. agalactiae* to colonise the mammary gland. This environment facilitates bacterial attachment and survival in mammary tissues due to the high Ca^2+^ content of milk [45].

Fimbriae play an important role in GBS adherence, biofilm formation and host specificity, and three pilus islands, PI-1, PI-2a and PI-2b, have been identified, which encode distinct pilus structures that mediate interactions with host cells [46,47]. A study analyzing 295 different strains from multiple sources in North America found that most bovine strains had only PI-2b, a feature also observed in bovine strains from other geographic locations [48]. The differences in PI frequencies between bovine and human strains suggest that a type of fungus (mycorrhiza) contributes to host specificity.

In addition, the Sip protein is another key factor in invasion infections of *Strep. agalactiae*. As a highly conserved surface protein, it induces cross-immune protection and is widely present in the bacterium. This makes it a promising candidate for use in the preparation of mastitis-derived *Strep. agalactiae* subunit vaccines for dairy cows [49].

### 3.2. Exotoxins

The CAMP factor is an extracellular protein that leads to the formation of pores in target cells, and has been extensively used in the identification of streptococci of mastitis origin [50]. In addition, vaccines based on the immunogenic activity of the CAMP factor to confer protective immunity against *Strep. agalactiae* in mice and cows have been developed [51,52].

### 3.3. Others

Hyaluronidase, encoded by *hylB*, promotes the entry of *Strep. agalactiae* into host cells and has a strong role in stimulating the secretion of pro-inflammatory cytokines, as well as a trophic role [53]. *Strep. agalactiae* hyaluronidase degrades host hyaluronan into its disaccharide components, which are immunosuppressive because they bind to TLR2/TLR4 receptors and block signaling [54]. The *hylB* gene is frequently detected in mastitis-derived *Strep. agalactiae* strains in dairy cows, showing that hyaluronidase plays an important role in the infection process [55].

## 4. Environmental Streptococci and Enterococci

### 4.1. Streptococcus uberis

**Figure 5:**
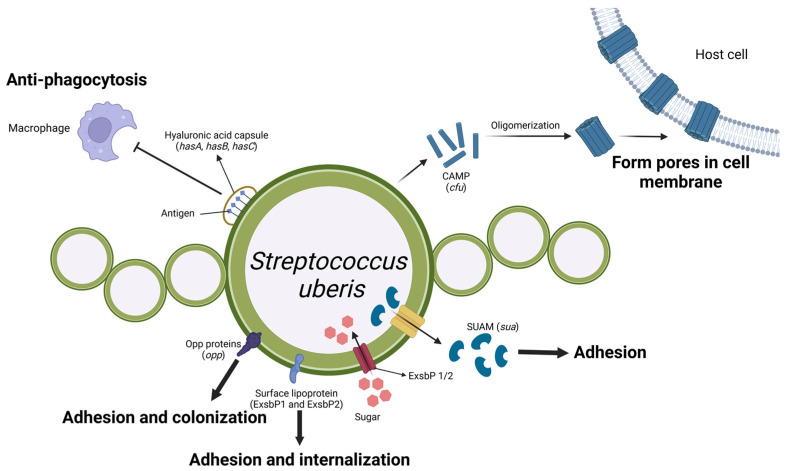
Schematic representation of *Streptococcus uberis* virulence factors and their roles in mastitis pathogenesis. The hyaluronic acid capsule, encoded by the *hasA*, *hasB*, and *hasC* genes, protects the surface antigens of *Strep. uberis* from phagocytosis by macrophages. *Strep. uberis* secretes monomer CAMP factors encoded by the *cfu* gene, which oligomerizes to form tube-like structures that create pores in the host cell membrane, ultimately causing cell damage. The Opp proteins, surface lipoprotein (Slp), and SUAM protein, encoded by the *opp*, *slp*, and *sua* genes, respectively, facilitate bacterial adhesion and colonisation on host cells. Furthermore, the ExsbP1/2 proteins function as transporters of sugars from the external environment into the bacteria. (Created with BioRender.com).

Several virulence factors of *Strep. uberis* have been identified, including acid capsules, hyaluronidase, plasminogen activator proteins, *Strep. uberis* adhesion molecule (SUAM), Opp proteins, and elongation factor Ts (Figure 5). Genetic differences in certain virulence factors among strains lead to variations in their pathogenic potential. Aureema et al. (2019) distinguished between mastitis-associated and unassociated *Strep. uberis* isolates through the creation of a subtractive diversity array (SDA) focused on virulence determinants, which highlights the specific role of certain virulence factors in the pathogenic process [56]. In addition, other researchers using MLST [57], experimental challenge [58], microarray analysis [59], and PFGE [60] have concluded that specific *Strep. uberis* strains are suitable for spreading among dairy cows and can cause mastitis, while other strains act mainly as environmental strains. Therefore, virulence-associated factors of the strains appear to be an important determinant of pathogenicity.

Since *Strep. uberis* predominantly colonizes the ducts, tissues, and secretory alveolar lumens and rarely invades mammary tissues, the corresponding gene products may be important for the growth or survival of *Strep. uberis* within the bovine mammary glands [61]. Taylor et al. (2003) identified several metabolism-related genes that play a crucial role in *Streps uberis*’s growth and survival [62]. The discovered genes, particularly those encoding proteins involved in sugar metabolism, were upregulated during in vitro growth in milk. These metabolic genes may contribute to mastitis development by enhancing the uptake of sugars and peptides, thereby facilitating bacterial growth in milk. Notably, whole-genome sequencing also identified several key metabolic genes in *Strep. uberis*. These genes are critical for the growth and survival of *Strep. uberis* in the mammary gland environment [63]. These genes include a putative fructan beta-fructosidase precursor, which is involved in the breakdown of fructan for bacterial utilization and is particularly important for energy production and cell wall synthesis. Additionally, the lactoferrin binding protein, although mainly involved in obtaining iron by binding lactoferrin, is essential for bacterial metabolic activity and growth and reproduction, especially under iron-restricted conditions (e.g., during the early dry period when iron is bound to lactoferrin, or during early lactation, characterized by elevated lactoferrin levels and reduced iron bioavailability) [64]. The expression patterns and variations in these genes may critically influence the pathogenicity and adaptability of *Strep. uberis*.

In recent years, there has been significant interest in the development of vaccines against *Strep. uberis*, with a particular focus on proteins that exhibit potent immunogenic properties. According to current studies, promising antigenic candidates for the *Strep. uberis* bovine mastitis vaccine include fructose-bisphosphate aldolase (FBA), surface lipoproteins (SLPs), and extracellular sugar-binding proteins 1 and 2 (ExsbP1 and ExsbP2) [65,66]. In fact, a commercial vaccine, UBAC^®^, has been developed to prevent *Strep. uberis* infections in cattle. This vaccine is specifically designed to reduce the incidence of clinical mastitis caused by *Strep. uberis* and has garnered attention for its effectiveness in lowering SCC in milk. UBAC^®^ is a subunit vaccine that incorporates lipoteichoic acid, a crucial virulence factor of *Strep. uberis* and an essential component of the Gram-positive bacterial cell wall [67]. In vivo studies have shown that UBAC^®^-vaccinated cattle exhibited milder clinical signs after experimental unrelated strain *Strep. uberis* infection, and milk production recovered more quickly in the vaccinated herd, highlighting the vaccine’s potential benefits [67]. In addition, vaccinated cows had a reduced increase in body temperature post-infection compared to the control group, suggesting that the vaccine may attenuate the systemic inflammatory response associated with the infection [67]. UBAC^®^-vaccinated cows had significantly fewer clinical signs, bacterial counts, rectal temperatures, and daily milk production losses following intramammary infection, and significantly increased the number of quarters with no bacterial isolation and SCC below 200,000 cells/mL at the end of the study [67]. These results provide ample evidence supporting the vaccine’s efficacy and underscore its potential utility in the prevention of bovine mastitis.

The evolving understanding of the *hasABC* operon in *Strep. uberis* highlights the complexity of bacterial virulence. Early research by Ward et al. (2001) emphasized the role of hyaluronic acid capsules in antiphagocytosis, proposing that the *hasABC* gene is critical for virulence [68]. However, subsequent studies, particularly those by Field et al. (2003), challenged this notion by demonstrating the retained virulence of *hasA* mutants, indicating the presence of compensatory pathways that may sustain capsule-independent infections [69]. Further investigations by Srithanasuwan et al. (2022) revealed strain-specific differences in virulence, with strains lacking the *hasABC* gene, essential for envelope synthesis, being linked to transient intramammary infections (IMIs). This suggests that *Strep. uberis* may adapt to diverse environmental pressures through distinct genetic mechanisms [70]. These findings suggest that, although *hasABC* manipulators are significant, *Strep. uberis* exhibits a multitude of pathogenic traits that are shaped by broader genomic contexts and environmental interactions, emphasizing the necessity for ongoing research into its pathogenic mechanisms.

Notably, the finding by Srithanasuwan et al. (2022) that the *cfu* gene is present in transient *Strep. uberis* IMIs is consistent with previous findings that “non-hemolytic streptococcal strains were able to survive in significantly higher numbers than hemolytic streptococci” [70]. This competitive survival advantage may explain why non-hemolytic streptococci are more likely to cause persistent mastitis, while hemolytic streptococci are more likely to cause transient mastitis. However, it is important to note that studies on the hemolytic properties of *Strep. uberis* in the mammary environment are relatively limited. Therefore, the exact relationship between virulence factors and mammary immune responses still requires further in-depth studies and validation.

### 4.2. Streptococcus dysgalactiae

Unlike *Strep. agalactiae* and *Strep. uberis*, most *Strep. dysgalactiae* strains are CAMP-negative, do not catabolize aesculin and are usually classified as Lancefield C group [71]. *Strep. dysgalactiae* infects mainly the bovine mammary tissue and contains virulence factors similar to *Strep. agalactiae* and *Strep. uberis*, including α-C protein, β-C protein, hyaluronidase, C5a peptidase, and glyceraldehyde-3-phosphate dehydrogenase [72,73]. These virulence factors assist the bacteria to adhere to bMECs and invade cells [74]. In addition, mitogenic superantigens derived from *Strep. dysgalactiae* are virulence factors that mediate immune escape and can lead to overactivation of the immune system, thereby inducing inflammation [75,76].

### 4.3. Enterococcus spp.

Virulence factors of *Enterococcus* spp., including *Enterococcus faecalis* and *Enterococcus faecium*, have been extensively studied in other host species (e.g., humans or experimental animals) using non-mammary infection models. These factors include the enterococcal surface protein (*esp*), gelatinase (*gelE*), and cytolysin (*cyl*). Genes encoding these virulence factors were often detected in *Enterococcus* spp., causing mastitis in dairy cows. However, their role and significance in *Enterococcus* spp.-induced bovine mastitis has not been studied. More research is needed to determine their relevance and contribution to mastitis in dairy cows.

## 5. *Escherichia coli*

**Figure 6:**
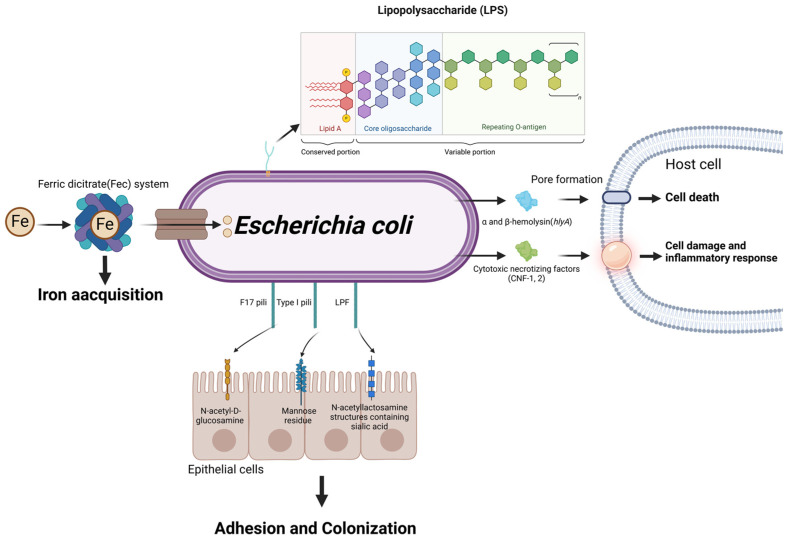
Schematic representation of *Escherichia coli* virulence factors and their roles in mastitis pathogenesis. Through the ferric dicitrate system, *E. coli* acquires Fe from the external environment, which is crucial for its growth and proliferation. The adherence and colonization of host tissues are facilitated by various types of pili, including Type I, F17 and LPF pili, which interact with specific receptors on host epithelial cells. Subsequently, lipopolysaccharides (LPSs) and α/β-hemolysin collaborate to form pores in the host cell membrane, resulting in cell death. Furthermore, CNF-1 intensifies cell damage and inflammation, contributing to the destruction of cellular structures and the development of pathogenic inflammation. (Created with BioRender.com).

*E. coli* is prevalent in dairy farm environments and is frequently identified as a pathogen causing mastitis in dairy cows. Mastitis caused by *E. coli* typically manifests as an acute infection with severe clinical signs [77]. *E. coli* is highly diverse and is classified into many pathogenic types or variants based on virulence factor pool, clinical signs, and infection site [78]. The term mammary pathogenic *E. coli* (MPEC) has been proposed to describe *E. coli* strains able to infect the mammary gland [79]. Current research focuses on virulence factors associated with bovine mastitis including lipopolysaccharides, exotoxins, adhesins, and iron carriers (Figure 6). However, Kempf et al. (2016) [80] showed that *E. coli* strains responsible for bovine mastitis typically lack many of the virulence genes commonly found in other pathogenic *E. coli* strains. In particular, they noted that *E. coli* strains causing mastitis lack unique genes associated with lipopolysaccharide (LPS) synthesis, iron acquisition, and type 6 secretion systems, which are common virulence factors in other *E. coli* pathogens. MPEC are also diverse, and to date, no specific virulence factors or clusters of virulence mechanisms have been definitively linked to mammary pathogenicity.

### 5.1. Adherence

Adhesion of *E. coli* to host cells is mediated by binding to fibrin and lamin receptors on host cells [81]. Pili and non-pili adhesins play a key role in *E. coli* colonisation (Figure 6). Among them, the *f17A* gene encoding F17 fimbriae is the most prevalent virulence factor in pathogenic *E. coli* isolated from dairy mastitis cases [82]. In addition, a study in which the *f17A* gene was cloned and prokaryotically expressed from *E. coli* isolates from dairy cows with mastitis found that the F17A protein was able to trigger a protective immune response, supporting its potential as a vaccine candidate antigen [83]. A recent study showed that long polar fimbriae (LPF) plays a crucial role in the colonization of bMECs by MPEC, potentially facilitating the bacteria’s long-term survival and contributing to chronic infections [84].

In addition to these adhesins, the high frequency of ecpA and fimH in clinical isolates of *E. coli* with dairy mastitis further emphasizes their significance in bacterial adhesion and immune escape [85]. Specifically, *ecpA* encodes an adhesin involved in the early stages of biofilm formation, while *fimH* encodes type I fimbriae that mediates bacterial attachment to epithelial cells through mannosy-binding mechanisms [85,86].

### 5.2. Exotoxins

Hemolysin and cytotoxic necrotizing factor (CNF) toxins (CNF1 and CNF2) are well-known exotoxins and the genes encoding these exotoxins have been detected in dairy-derived *E. coli* [87,88]. According to Sun et al. (2021) a hemolysin-deficient strain of *E. coli* from mastitis was constructed and found to be considerably less virulent [89]. This suggests that in *E. coli* that can express hemolysin, hemolysin output is necessary for it to exert its full virulence. However, hemolysin was detected in less than 10% of infected dairy cows with mastitis [85,90], suggesting that hemolysin may not be a dominant virulence factor in infected dairy cows with mastitis. However, in conjunction with the findings of Sun et al. (2021) [89], perhaps the presence of hemolysin confers an additional virulence mechanism to the pathogen during infection, which requires further study of the differences in pathogenicity between hemolysin-expressing and non-hemolysin-expressing *E. coli* from mastitis-derived *E. coli*.

Two types of cytotoxic necrosis factor (CNF) have been identified, one CNF1 and the other CNF2. Sun et al. (2021) constructed CNF2 deletion mutants for in vivo and in vitro experiments, and found that the CNF2 deletion mutants showed a significant reduction in virulence, a result that suggests that CNF2 toxin export is required for the full virulence of the mastitis strain BCE049 bacteria [89]. Currently, the *cnf2* gene has never been found in human extraintestinal pathogenic *E. coli* (ExPEC). Instead, CNF2 appears to be prevalent in strains isolated from neonatal calves suffering from extraintestinal infections, which appears to be its close association with disease in cattle (calves and dairy cows) [91].

### 5.3. Endotoxin

LPS is a major component of the outer membrane of Gram-negative bacteria (*E. coli*, *K. pneumoniae*). It elicits an inflammatory response that leads to increased expression of TNF-α, IL-6, and IL-1β in mammary tissue and bMECs [92]. LPS consists of three regions: lipid A, core oligosaccharide, and O-antigen. Interestingly, several lipid A-modifying enzymes have been described that, if present, can modulate the innate immune response in dairy cows [93]. However, it has been shown that such lipid A-modifying enzymes are not present in mastitis isolates [80]. Furthermore, *E. coli* can be categorized into five different core types based on the structure of the core oligosaccharides. Currently reported isolates of mastitis-derived *E. coli* have K-12, R2, or R4 core types, and it is not yet possible to conclude the existence of a preferred core oligosaccharide subtype for mastitis [80,94]. As for the O-antigen, previous results have shown that specific serotypes are not associated with mastitis isolates [95].

### 5.4. Siderophore

A pivotal trait facilitating the adaptation and proliferation of *E. coli* in mammary tissue is its capacity to acquire iron from milk. Due to the low solubility of iron, highly affine iron transport systems are essential. *E. coli* expresses various iron transport systems, such as enterobactin, aerobactin, ferrichrome, and iron-regulated outer membrane proteins (IROMPs), which bind to ferric siderophore complexes and facilitate iron uptake into the bacterial cells. Notably, the ferric dinitrate (*Fec*) system of *E. coli* demonstrates a strong iron scavenging capability, closely associated with mastitis-related *E. coli* strains [96,97,98]. The *Fec* system is crucial for the growth of *E. coli* in milk, and significantly affects the severity of mastitis infections [99]. Population-level genomic analysis reveals that the *Fec* system is vital for the pathogenicity of *E. coli* in the mammary environment [96]. Studies show that typical mastitis-causing *E. coli* strains (MPEC) P4 cannot induce mastitis in the absence of *Fec*, while non-mastitis-inducing strains, such as the K71 strain, gain MPEC capabilities upon the acquisition of *Fec* [100]. This further suggests that the *Fec* system is likely essential for growth in milk, potentially reducing the dependence on additional iron acquisition genes. This may explain the lower prevalence rates (3.5–26.4%) of other ferric siderophore genes, such as *irp2*, *iucD*, *ireA*, and *sitA* in mastitis isolates [85,101].

## 6. *Klebsiella pneumoniae*

**Figure 7:**
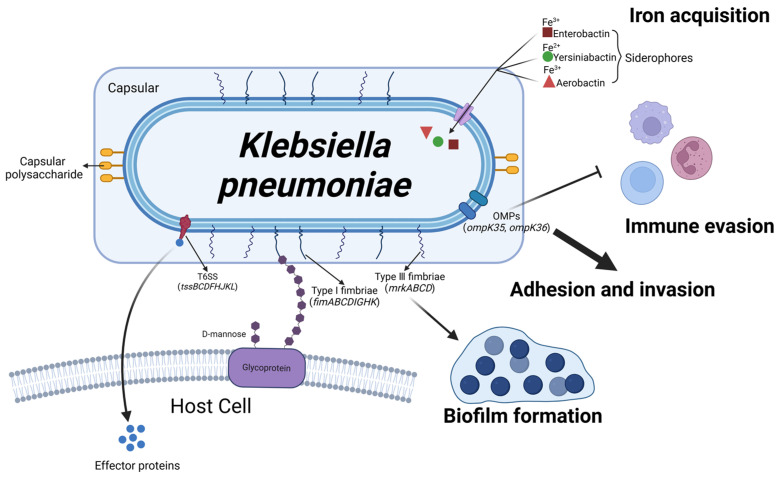
Schematic representation of *Klebsiella pneumoniae* virulence factors and their roles in mastitis pathogenesis. The serotypes of *K. pneumoniae* are determined by the capsular polysaccharides on its surface, enhancing its resistance to phagocytosis. Outer membrane proteins (OMPs), encoded by genes *ompK35* and *ompK36*, enable *K. pneumoniae* to evade the host immune system, while T6SS translocates effector proteins into target cells. Siderophores, such as enteroactin, yersiniabactin, and aerobactin, facilitate iron uptake (Fe) by binding to distinct iron sources. Upon leaving the host, *K. pneumoniae* can form biofilms through Type I and III pili, which prolong its survival until it reencounters a bovine host. (Created with BioRender.com).

*K. pneumoniae*, a Gram-negative bacterial species, is widely distributed on dairy farms, mainly in bedding, drinking water, milking machines, cow feces, and teat skin [77,102]. *K. pneumoniae*-induced mastitis is characterized by a rapid onset, the potential to elicit severe clinical signs, a diminished response to antimicrobial therapy, and a high mortality rate. Additionally, it leads to a substantial decrease in milk yield, resulting in significant economic losses for dairy farms [102]. The pathogenicity of *K. pneumoniae* is related to its virulence factors, with the primary factors being capsular polysaccharide (CPs), lipopolysaccharides, siderophores, and fimbriae (Figure 7).

### 6.1. Capsular Polysaccharide

CP exists on the bacterial surface and is involved in bacterial–host interactions. According to the CP antigen structure, a total of 79 capsular types have been identified, from which K1, K2, K5, K54 and K57 are considered as hypervirulent *K. pneumoniae* (hvKp) strains [103,104]. In China, the prevalence of K57 strains was significantly higher than that of other highly virulent capsular serotypes [104]. Additionally, K57 strains were more prevalent in milk from clinical mastitis than in other sources on farms [105]. Kanevsky-Mullarky et al. (2014) reported that *K. pneumoniae* derived from chronic mastitis was able to form a thicker capsule and exhibited increased resistance to phagocytosis by bovine polymorphonuclear leukocytes compared to other strains [106].

### 6.2. Adherence

In the pathogenesis of *K. pneumoniae*, fimbriae are recognized as pivotal mediators of adhesion to host cells [107]. Adhesion, being the inaugural step in pathogen colonization and infection, underscores the significance of fimbriae in the infection process [108]. *K. pneumoniae* possesses two primary fimbrial structures: type I and type III fimbriae [108]. Type I fimbriae is homologous to *E. coli* and is encoded by a suite of genes such as *fimABCDGIH* [108]. In contrast to *E. coli*, *K. pneumoniae* uniquely possesses the *fimK* gene, the function of which, though not fully elucidated, is postulated to significantly influence the regulation of type I fimbriae expression. Studies have shown that the absence of fimK may result in the loss of type I fimbriae, thereby weakening the strain’s adhesion capability [109]. Type I fimbriae can bind to D-mannosylated glycoproteins, and are therefore known as “mannose-sensitive” fimbriae [110]. In contrast, type III fimbriae are encoded by the *mrkABCD* gene cluster and have a helical structure that does not bind to mannose and are therefore known as “mannose-insensitive” fimbriae [108]. Type III fimbriae are often associated with biofilm formation and long-term colonization, especially in urinary tract infections and airway infections [111,112]. The detection rates of the *fimH* gene associated with type I fimbriae and the *mrkD* gene associated with type III fimbriae in clinical mastitis isolates exceeded 80% [105]. Although the role of these fimbriae in dairy mastitis is currently unclear, fimbriae have been shown to promote the adhesion of *K. pneumonica* to different epithelial cells (such as urethral epithelial cells, bladder epithelial cells and trachea epithelial cells) [113], so it is speculated that the adhesion function of fimbriae in bMECs may be equally important, and further studies are needed to verify it.

In addition, a recent study using a clinical isolate of dairy mastitis to vaccinate with recombinant FimA, FimC, and FimG proteins showed that these proteins triggered a significant humoral immune response in mice, with FimG exhibiting exceptional efficacy in safeguarding against *K. pneumoniae* mastitis [114]. This finding posits FimG as a potential candidate for the development of a vaccine to prevent *K. pneumoniae* mastitis. However, the study used intraperitoneal injection of bacteria to simulate infection, which does not directly reflect the natural infection route of mastitis. Therefore, these findings need to be further validated in models that more closely resemble the actual pathological course of mastitis to assess their potential for clinical application.

### 6.3. Others

Iron is an essential element for the growth of *K. pneumoniae*. Studies have shown that *K. pneumoniae* isolated from clinical mastitis, which possess siderophore genes, exhibits enhanced growth in iron-deficient environments [115]. The iron acquisition proteins related to these siderophore genes are highly conserved among various bacteria and are widely expressed during infection, presenting them as promising targets for vaccine development. In the United States, vaccines containing the siderophore receptor and porin protein (SRP) have been developed for the prevention of mastitis caused by *K. pneumoniae* [116].

Whole genome sequencing studies have revealed the genetic diversity of *K. pneumoniae* among different hosts, especially between bovine mastitis-derived strains and other source strains. Holt et al. (2015) found that lactose operon (*lac*) was carried in higher amounts in the bovine mastitis-derived strain than in the human strain, suggesting that it could enhance the aggressiveness of *K. pneumoniae* in cow mammary tissue [117]. Yang et al. (2019) reported a higher likelihood of bovine *K. pneumoniae* isolates to harbor genes encoding metal ion proteins, hypothesizing that the iron uptake protein-encoding operon (*kfu*) contributes to the pathogenesis of bovine mastitis [118]. Zheng et al. (2022) reported that virulence factors associated with ferric citrate uptake, lactose fermentation, and heavy metal resistance were detected at significantly higher levels in mastitis-derived strains than in strains from other sources [119]. While these genomic discoveries have provided valuable insights into possible genetic bases and pathogenic characteristics, current research has mostly remained at the level of genomic analysis. Therefore, the function of these genes and their specific contribution to pathogenicity require further validation through comprehensive experimental studies. In particular, it is imperative to ascertain through experimental models whether differences in the carrying rates of these genes directly affect the pathogenicity of dairy mastitis-derived *K. pneumoniae*.

## 7. Prospects

Going forward, there is a need for further research on the virulence factors of mastitis-causing bacteria in dairy cows, especially more in vivo studies or studies targeting bMECs. In light of this, the following research orientations have been pointed out for a comprehensive understanding of the virulence factors of mastitis-causing bacteria in dairy cows.

### 7.1. Comparative Analysis of Virulence Factors in Mastitis-Causing Pathogens

Mastitis-derived pathogens in dairy cows carry different virulence factors that allow their pathogenicity to vary between genera, species and strains, and can lead to mastitis from mild to severe. Both *Staph. aureus* and *Strep. agalactiae* are biofilm-forming Gram-positive bacteria, but *Staph. aureus* infections are usually more persistent and more difficult to treat than *Strep. agalactiae*. This may be due to the relatively strong biofilm formation ability of *Staph. aureus* in mammary tissue. For example, the Bap protein of *Staph. aureus* cleaves into fragments in a low-calcium environment to form amyloid fibrils that provide a scaffold for biofilm development, and there is also a PIA-dependent manner of biofilm formation dominated by the ica locus, whereas *Strep. agalactiae* mediates biofilm formation mainly through pilus. Furthermore, *Staph. aureus* possesses a more complex toxin production system, enabling it to produce a greater variety of aggressive exotoxins, including enterotoxins, leukotoxins, and TSST-1. *Strep. agalactiae* and environmental streptococci (*Strep, uberis*, *Strep. dysgalactaie*, and *Enterococcus* spp.) belong to the same genus, whereas they have different modes of transmission. *Strep. agalactiae* mainly cause contagious mastitis, while environmental streptococci mainly cause environmental mastitis. This distinction may stem from differences in virulence factor expression, adhesion capacity, and immune evasion mechanisms between *Strep. agalactiae* and environmental streptococci. These differences result in their different pathogenic mechanisms and clinical manifestations of mastitis, making *Strep. agalactiae* more inclined to cause contagious mastitis and environmental streptococci more frequently to cause environmental mastitis. However, these observations remain speculative, as no studies have definitely elucidated the reasons for these variations. Further research is warranted to delve deeper into the virulence factors and pathogenic mechanisms of mastitis-causing bacteria in dairy cows, which could facilitate the development of more precise prevention and treatment strategies for mastitis.

### 7.2. Cross-Species Comparison of Virulence Factors in Mastitis Pathogens

It is noteworthy that mastitis pathogens in dairy cows could potentially pose a threat to human health at the species level, yet it is uncommon for the same strain to infect both humans and animals. With comparative genomic analysis examining the differences in virulence factors between infected cows and other animal isolates, the presence of distinct virulence factor signatures is promising. For instance, comparative genomic analysis of *K. pneumoniae* isolates from humans and mastitis-affected cows has revealed significant genomic structure disparities, including virulence determinants and resistance patterns, with a higher prevalence of genes related to metal ion metabolism and lactose manipulation in bovine isolates. However, in vitro and in vivo experiments are lacking to demonstrate whether the presence of these different virulence factors makes them more likely to cause mastitis in cows.

### 7.3. Exploring and Validating Novel Virulence Factors in Mastitis Pathogens

When examining the virulence factors of mastitis-causing bacteria in cows, it is beneficial to investigate if corresponding studies have been conducted in other species, as they may provide insights. To be specific, research in human endothelial cells has shown that the ClfA protein expressed by *Staph. aureus* inhibits phagocytosis by interacting with complement regulatory factor I, thereby mediating the cleavage of C3b in vivo. Whether this mechanism also exists in mastitis requires further validation. Furthermore, research on multiple virulence factors of enterococci has been limited to humans or other species. Meanwhile, there is a necessity to explore novel virulence factors, which can be achieved through preliminary genomic analysis aided by computational tools, or by employing gene prediction tools such as Prodigal, GeneMark, and Glimmer to identify potential coding regions. Subsequently, protein structure prediction can be performed for potential virulence factor genes. By integrating genomic, protein structure and function prediction results, in vivo and in vitro experimental validation is performed to uncover new virulence factors of mastitis-causing bacteria in cows.

### 7.4. Vaccine Development Based on Pathogen Virulence Factors

In an effort to protect cow health, improve dairy product quality, and reduce treatment costs, researchers have utilized virulence factors of pathogens to develop vaccines against bovine mastitis. However, most of them have been only tested in mouse experiments with limited application in dairy cows or on pasture. These vaccines primarily consist of recombinant subunit vaccines against *E. coli* virulence factor f17A, and *Strep. uberis* fructose-bisphosphate aldolase (FBA), surface lipoproteins (slp), exsbP1, and exsbP2 were shown to be immunogenic. In addition, a vaccine against *K. pneumoniae* has been developed that utilizes the bacterium’s siderophore receptor protein (SRP) as a key component. The commercially available vaccine for *Strep. uberis*, UBAC^®^, contains the key virulence factor lipophosphatidic acid (LTA), which, when immunized, shows the potential to reduce the incidence of clinical mastitis and lower SCC in milk.

## 8. Conclusions

The virulence factors of *Staph. aureus*, *E. coli*, and *Strep. agalactiae*, which are associated with bovine mastitis, have been the subject of extensive research. *Staph. aureus* has a well-established toxin production system that secretes various exotoxins (α,β,δ-hemolysin, leukotoxins, enterotoxins, and Toxic shock syndrome toxin). *E. coli*, implicated with mastitis, has been identified as a novel pathogenic type, with key virulence factors characterized by fimbriae and non-fimbriae adhesins, LPS, siderophore, and exotoxins (α and β-hemolysin, cytotoxic necrotizing factors, Shiga toxin). The virulence factors of *Strep. agalactiae* include proteins (fibrinogen binding protein and Rib protein), toxins (β-hemolysin/lecithin and CAMP factors), enzymes (C5a enzyme and hyaluronidase), with the presence of specific CAMP factors being diagnostic for this species. Despite a paucity of research on the virulence characteristics of environmental *Streptococcus* spp. and *K. pneumoniae*, the available data offer valuable insights. In contrast to *Strep. agalactiae*, environmental streptococci lack the FbsA and pilus virulence factors, and *Strep. dysgalactiae* and *Enterococcus* spp. do not possess CAMP factors. *K. pneumoniae* has similar virulence factors to *E. coli*, but it is less capable of secreting exotoxins than *E. coli*.

A comprehensive understanding of these bacteria’s virulence factors is essential for elucidating the pathogenesis of mastitis. While significant progress has been made, research is still needed on less-studied pathogens, including *Mycoplasma* spp., which is gaining importance in dairy industries of developed countries. This will facilitate the development of effective preventative and therapeutic strategies aimed at enhancing bovine health and ensuring dairy product safety.

## Data Availability

No new data were created or analyzed in this study.
